# Synthesis and crystal structure of calcium hydrogen phosphite, CaHPO_3_

**DOI:** 10.1107/S2056989019008235

**Published:** 2019-06-14

**Authors:** Mark L. F. Phillips, William T. A. Harrison

**Affiliations:** a Pleasanton Ridge Research LLC, Sandia Airpark, Edgewood, New Mexico 87015, USA; b Department of Chemistry, University of Aberdeen, Meston Walk, Aberdeen AB24 3UE, Scotland

**Keywords:** crystal structure, calcium, hydrogen phosphite

## Abstract

The hydro­thermal synthesis and crystal structure of the simple inorganic compound CaHPO_3_, which crystallizes in the chiral space group *P*4_3_2_1_2, are reported. The structure is built up from distorted CaO_7_ capped trigonal prisms and HPO_3_ pseudo pyramids, which share corners and edges to generate a three-dimensional network.

## Abstract 2

The title compound is built up from CaO_7_ polyhedra and HPO_3_ pseudo tetra­hedra sharing corners and edges to generate a three-dimensional network.

## Chemical context   

Calcium–phospho­rus–oxygen phases are ubiquitous in inorganic and materials chemistry. They have long been known as key components of fertilizers produced on a multi-million tonne scale (Rajan *et al.*, 1996 ▸) and more recently their clinical applications as cements and biomaterials have been intens­ively studied. A recent review (Eliaz & Metoki, 2017 ▸) refers to over 860 articles. Some of their other applications include use as additives in cheese making (Lucey & Fox, 1993 ▸), as environmental remediation agents (Nzihou & Sharrock, 2010 ▸) and as corrosion inhibitors (del Amo *et al.*, 1999 ▸). Apatite, Ca_5_(PO_4_)_3_ *X* (*X* = OH, F, Cl, ⋯), is the most abundant calcium phosphate mineral and is of great importance in mineralogy and geochemistry (Hughes & Rakovan, 2002 ▸).

As part of our ongoing exploratory synthetic studies, we now describe the hydro­thermal syntheses and crystal structure of CaHPO_3_, (I).

## Structural commentary   

**Figure 1:**
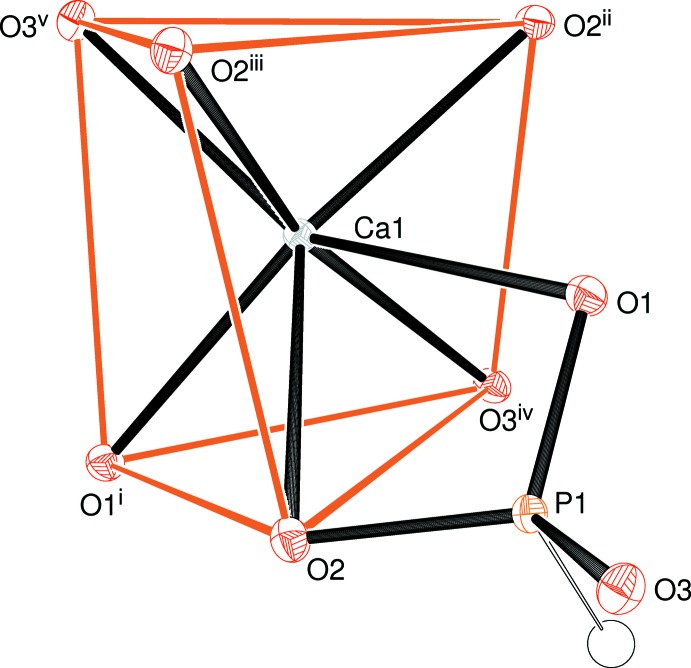
The asymmetric unit of (I) expanded to show the complete calcium coordination polyhedron (50% displacement ellipsoids). See Table 1 ▸ for symmetry codes.

The asymmetric unit of (I) has the simple composition of one Ca^2+^ cation and one hydrogen phosphite anion (Fig. 1 ▸) with the symmetry elements of the chiral tetra­gonal space group *P*4_3_2_1_2 building up the complete crystal structure. This results in the calcium ion being coordinated by seven O atoms belonging to six different HPO_3_^2−^ groups (the one in the arbitrarily chosen asymmetric unit is chelating *via* the O1,O2 edge). The Ca1—O2 distance of 2.6938 (14) Å (Table 1 ▸) is notably longer than the others (mean for the six shorter distances = 2.391 Å) but it clearly qualifies as a bond based on the Brown criterion (Brown, 2002 ▸) of contributing at least 0.04 v.u. (valence units) to the bond-valence sum (BVS; Brown & Altermatt, 1985 ▸) for the metal ion: the Ca1 BVS is 2.07 v.u. (expected value = 2.00 v.u.), with the long O2 bond contrib­uting 0.14 v.u. The next-nearest oxygen atom is some 3.68 Å distant from the calcium atom.

The calcium coordination environment in (I) can be described as a distorted mono-capped trigonal prism with O1^i^/O2/O3^iv^ and O2^ii^/O2^iii^/O3^v^ forming the ends of the prism and the Ca1—O1 bond protruding through the twisted rectangular face formed by atoms O2/O2^iii^/O2^ii^/O3^iv^ (see Table 1 ▸ for symmetry codes). The dihedral angle between the end-faces noted in the previous sentence is 6.90 (9)°, and the calcium ion is displaced from them by 1.5607 (8) and 1.4454 (9) Å, respectively. The dihedral angles subtended by O1^i^/O2/O3^iv^ and O1/O2/O3^iv^ (the latter being the triangle formed by the protruding atom O2 and the common edge with the prism-end) is 36.14 (7)°; the equivalent value for O2^ii^/O2^iii^/O3^v^ and O1/O2^ii^/O2^iii^ is 41.37 (4)°. These data, especially the second value, are in very good agreement with the ideal value of 41.5° for a capped trigonal prism with *C*_2*v*_ symmetry built up from hard spheres (Lewis & Lippard, 1975 ▸). Finally, it may be noted that the calcium ion is displaced from the centroid of its seven associated O atoms by 0.34 Å approximately away from the O1/O2 edge of the chelating phosphite group.

The HPO_3_^2–^ hydrogen phosphite group in (I) displays its normal (Loub, 1991 ▸) tetra­hedral (including the H atom) shape with a mean P—O separation of 1.526 Å. The bond to O2 is clearly longer than the others (Table 1 ▸), which might correlate with the fact that O2 bonds to three calcium cations, whereas O1 and O3 bond to two. The O—P—O angles are notably distorted with O1—P1—O2 (the chelating atoms to the adjacent calcium ion) some 8° smaller than the other two angles. The P atom is displaced by 0.4200 (9) Å from the plane of its attached O atoms, which is typical (Holmes *et al.*, 2018 ▸). As usual, the hydrogen atom of the phosphite group shows no propensity to form hydrogen bonds and in (I) atom H1 ‘points into space’ with its nearest neighbour being another H1 atom at 2.08 (4) Å. The mean P—O bond length in the ‘type e’ (isolated) HPO_3_^2–^ groups surveyed by Loub (1991 ▸) of 1.517 Å is slightly shorter than the value for (I) but the mean P—H separation of 1.30 Å established by Loub is identical to the refined value for (I).

Each of the three unique O atoms in (I) has a different coordination environment: O1 bonds to two Ca cations and one P atom in an approximate T-shape with Ca—O—Ca = 108.30 (5) and Ca—O—P = 99.88 (7) and 150.21 (8)°. The environment of O2 can be described as a distorted OPCa_3_ tetra­hedron [range of angles = 91.01 (6)–131.49 (8); mean = 107.0°] whereas O3 bonds to two Ca and one P atom in an approximate trigonal arrangement [Ca—O—Ca = 103.77 (5) and Ca—O—P = 127.66 (7) and 128.07 (7)°; bond-angle sum = 359.5°].

**Figure 2:**
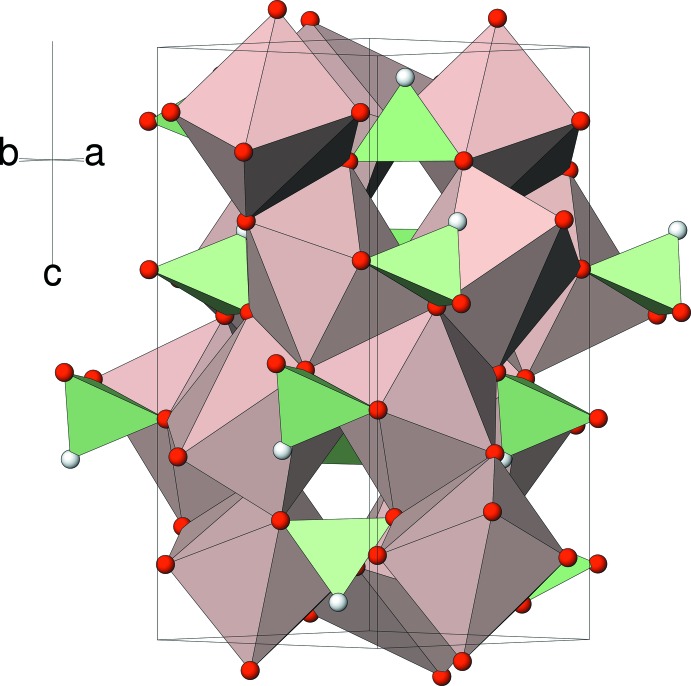
Polyhedral view of the packing in (I) viewed down [110].

The extended structure of (I) in polyhedral representation is shown in Fig. 2 ▸. The linkage of the CaO_7_ and HPO_3_ polyhedra generates a dense three-dimensional network in which each calcium cation is surrounded by five others linked *via* edges with Ca⋯Ca separations clustered in the narrow range of 3.6938 (5)–3.8712 (5) Å. There appear to be small voids in the structure but these correspond to the P—H vertices and a *PLATON* (Spek, 2009 ▸) analysis did not reveal any free space in the structure.

## Database survey   

A survey of the Inorganic Crystal Structure Database (ICSD; Belsky *et al.*, 2002 ▸), updated to March 2019, for compounds containing Ca, P, O and H and no other elements revealed 142 matches. The vast majority of these are phosphates (containing tetra­hedral P^V^O_4_ groups) and many of them are apatite derivatives. When the presence of any other element alongside Ca/P/O/H was allowed in the search, no fewer than 554 hits arose.

The closest analogues to (I) are the P^III^-containing phases calcium bis­(di­hydrogen phosphite) monohydrate, Ca(H_2_PO_3_)_2_·H_2_O [reported first by Larbot *et al.* (1984 ▸) (ICSD reference 36285) and then by Mahmoudkhani & Langer (2001*a* ▸; ICSD 280575) and calcium hydrogen phosphite monohydrate, CaHPO_3_·H_2_O (Mahmoudkhani & Langer, 2001*b* ▸; ICSD 411737). The water mol­ecule coordinates to the calcium cation in both compounds and O—H⋯O hydrogen bonds (from the OH moiety of the H_2_PO_3_ group and the water mol­ecule in 280575 and from the water mol­ecule in 411737) are prominent features of the crystal structures. It is notable that both phases feature a CaO_7_ coordination polyhedron with one chelating phosphite group: in 411737 its distorted capped trigonal–prismatic shape is similar to that seen in (I) whereas in 280575 it is closer to a penta­gonal bipyramid. The overall topology of the Ca/P/O bonding network in 411737 is layered but in 280575 it is three-dimensional.

## Synthesis and crystallization   

A mixture of 2.36 g (10.0 mmol) Ca(NO_3_)_2_·4H_2_O, 0.52 g (6.0 mmol) H_3_PO_3_ and 0.47 g (4.0 mmol) NH_4_ClO_4_ were dissolved in 10 ml H_2_O then mixed with 4.0 g 15 N NH_4_OH and loaded into a 23 ml Teflon cup. This was heated in a stainless steel pressure vessel for seven days at 473 K and cooled to room temperature over a few hours. Product recovery by vacuum filtration and rinsing with deionized water yielded 0.67 g (5.6 mmol; 93% yield based on Ca) of sparkling colourless prisms of (I).

A calculated X-ray powder pattern for (I) based on the single-crystal structure model was found to be in excellent agreement with its measured powder pattern (see supporting information): no ‘hits’ were found in a search against the JCPDS database of powder patterns. ATR–FTIR (diamond window, cm^−1^) for (I): 2467*w*, 2436*m* (P—H stretch); 1151*s*, 1056*vs*, 979*vs*, 588*vs*, 511*s*, 448*s* (phosphite P—O stretches and bends) [for the spectrum, see supporting information; for peak assignments, see Fridrichová *et al.* (2012 ▸)].

## Refinement   

Crystal data, data collection and structure refinement details are summarized in Table 2 ▸. Atom H1 was located in a difference map and its position and *U*_iso_ value were freely refined. The absolute structure of the crystal chosen for data collection is well-defined in space group *P*4_3_2_1_2 (No. 96) although the bulk sample presumably also consists of equal amounts of the other enanti­omer (space group *P*4_1_2_1_2, No. 92).

## Supplementary Material

Crystal structure: contains datablock(s) I, global. DOI: 10.1107/S2056989019008235/wm5511sup1.cif

Structure factors: contains datablock(s) I. DOI: 10.1107/S2056989019008235/wm5511Isup2.hkl

X-ray powder pattern and IR spectrum. DOI: 10.1107/S2056989019008235/wm5511sup3.docx

CCDC reference: 1921630

Additional supporting information:  crystallographic information; 3D view; checkCIF report

## Figures and Tables

**Table 1:** Selected bond lengths (Å)

Ca1—O1^i^	2.2870 (13)	Ca1—O2	2.6938 (14)
Ca1—O2^ii^	2.3600 (14)	P1—O1	1.5173 (13)
Ca1—O2^iii^	2.3907 (13)	P1—O3	1.5192 (13)
Ca1—O3^iv^	2.4014 (12)	P1—O2	1.5423 (13)
Ca1—O3^v^	2.4192 (13)	P1—H1	1.30 (2)
Ca1—O1	2.4869 (14)		

Symmetry codes: (i) 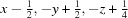; (ii) 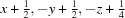; (iii) 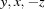; (iv) 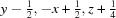; (v) 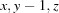.

**Table 2:** Experimental details

Crystal data	
Chemical formula	CaHPO_3_
*M*_r_	120.06
Crystal system, space group	Tetragonal, *P*4_3_2_1_2
Temperature (K)	100
*a*, *c* (Å)	6.67496 (6), 12.9542 (2)
*V* (Å^3^)	577.18 (1)
*Z*	8
Radiation type	Mo *K*α
μ (mm^−1^)	2.49
Crystal size (mm)	0.20 × 0.11 × 0.10

Data collection	
Diffractometer	Rigaku AFC12 CCD
Absorption correction	Multi-scan (*CrysAlis PRO*; Rigaku, 2017 ▸)
*T*_min_, *T*_max_	0.811, 1.000
No. of measured, independent and observed [*I* > 2σ(*I*)] reflections	7615, 668, 667
*R*_int_	0.030
(sin θ/λ)_max_ (Å^−1^)	0.649

Refinement	
*R*[*F* ^2^ > 2σ(*F* ^2^)], *wR*(*F* ^2^), *S*	0.014, 0.037, 1.27
No. of reflections	668
No. of parameters	51
H-atom treatment	All H-atom parameters refined
Δρ_max_, Δρ_min_ (e Å^−3^)	0.28, −0.38
Absolute structure	Flack *x* determined using 230 quotients [(*I* ^+^)−(*I* ^−^)]/[(*I* ^+^)+(*I* ^−^)] (Parsons *et al.*, 2013 ▸)
Absolute structure parameter	−0.006 (16)

Computer programs: *CrysAlis PRO* (Rigaku, 2017 ▸), *SHELXS97* (Sheldrick, 2008 ▸), *SHELXL2014* (Sheldrick, 2015 ▸), *ORTEP-3 for Windows* (Farrugia, 2012 ▸) and *publCIF* (Westrip, 2010 ▸).

## supplementary crystallographic information

### Crystal data

**Table:**

CaHPO_3_	*D*_x_ = 2.763 Mg m^−^^3^
*M**_r_* = 120.06	Mo *K*α radiation, λ = 0.71073 Å
Tetragonal, *P*4_3_2_1_2	Cell parameters from 6820 reflections
*a* = 6.67496 (6) Å	θ = 3.0–27.5°
*c* = 12.9542 (2) Å	µ = 2.49 mm^−^^1^
*V* = 577.18 (1) Å^3^	*T* = 100 K
*Z* = 8	Prism, colourless
*F*(000) = 480	0.20 × 0.11 × 0.10 mm

### Data collection

**Table:**

Rigaku AFC12 CCD diffractometer	668 independent reflections
Confocal mirrors, HF Varimax monochromator	667 reflections with *I* > 2σ(*I*)
Detector resolution: 28.5714 pixels mm^-1^	*R*_int_ = 0.030
ω scans	θ_max_ = 27.5°, θ_min_ = 3.4°
Absorption correction: multi-scan (*CrysAlis PRO*; Rigaku, 2017)	*h* = −8→8
*T*_min_ = 0.811, *T*_max_ = 1.000	*k* = −8→8
7615 measured reflections	*l* = −16→16

### Refinement

**Table:**

Refinement on *F*^2^	All H-atom parameters refined
Least-squares matrix: full	*w* = 1/[σ^2^(*F*_o_^2^) + (0.0206*P*)^2^ + 0.1946*P*] where *P* = (*F*_o_^2^ + 2*F*_c_^2^)/3
*R*[*F*^2^ > 2σ(*F*^2^)] = 0.014	(Δ/σ)_max_ < 0.001
*wR*(*F*^2^) = 0.037	Δρ_max_ = 0.28 e Å^−^^3^
*S* = 1.26	Δρ_min_ = −0.38 e Å^−^^3^
668 reflections	Extinction correction: SHELXL2014 (Sheldrick, 2015), Fc^*^=kFc[1+0.001xFc^2^λ^3^/sin(2θ)]^-1/4^
51 parameters	Extinction coefficient: 0.056 (4)
0 restraints	Absolute structure: Flack *x* determined using 230 quotients [(*I*^+^)-(*I*^-^)]/[(*I*^+^)+(*I*^-^)] (Parsons *et al.*, 2013)
Primary atom site location: structure-invariant direct methods	Absolute structure parameter: −0.006 (16)
Hydrogen site location: difference Fourier map	

### Special details

**Table:**

Geometry. All esds (except the esd in the dihedral angle between two l.s. planes) are estimated using the full covariance matrix. The cell esds are taken into account individually in the estimation of esds in distances, angles and torsion angles; correlations between esds in cell parameters are only used when they are defined by crystal symmetry. An approximate (isotropic) treatment of cell esds is used for estimating esds involving l.s. planes.

### Fractional atomic coordinates and isotropic or equivalent isotropic displacement parameters (Å^2^)

**Table:**

	*x*	*y*	*z*	*U*_iso_*/*U*_eq_	
Ca1	0.31831 (5)	0.10351 (5)	0.11917 (3)	0.00616 (14)	
P1	0.29735 (7)	0.57084 (7)	0.10542 (3)	0.00575 (15)	
H1	0.220 (4)	0.625 (4)	0.1928 (19)	0.013 (6)*	
O1	0.4785 (2)	0.43959 (19)	0.12612 (11)	0.0080 (3)	
O2	0.1356 (2)	0.4401 (2)	0.05361 (10)	0.0078 (3)	
O3	0.3393 (2)	0.76729 (19)	0.05029 (9)	0.0081 (3)	

### Atomic displacement parameters (Å^2^)

**Table:**

	*U*^11^	*U*^22^	*U*^33^	*U*^12^	*U*^13^	*U*^23^
Ca1	0.0058 (2)	0.00652 (19)	0.0061 (2)	−0.00010 (12)	0.00026 (13)	0.00022 (12)
P1	0.0057 (2)	0.0056 (2)	0.0059 (2)	−0.00049 (15)	−0.00009 (17)	0.00001 (15)
O1	0.0070 (6)	0.0078 (5)	0.0092 (5)	0.0004 (5)	−0.0005 (5)	0.0000 (5)
O2	0.0070 (6)	0.0086 (6)	0.0079 (5)	−0.0012 (5)	−0.0002 (5)	−0.0001 (5)
O3	0.0100 (6)	0.0067 (6)	0.0076 (6)	−0.0003 (5)	−0.0002 (5)	0.0005 (5)

### Geometric parameters (Å, º)

**Table:**

Ca1—O1^i^	2.2870 (13)	P1—O3	1.5192 (13)
Ca1—O2^ii^	2.3600 (14)	P1—O2	1.5423 (13)
Ca1—O2^iii^	2.3907 (13)	P1—H1	1.30 (2)
Ca1—O3^iv^	2.4014 (12)	O1—Ca1^ii^	2.2869 (13)
Ca1—O3^v^	2.4192 (13)	O2—Ca1^i^	2.3600 (14)
Ca1—O1	2.4869 (14)	O2—Ca1^iii^	2.3907 (13)
Ca1—O2	2.6938 (14)	O3—Ca1^vi^	2.4014 (12)
P1—O1	1.5173 (13)	O3—Ca1^vii^	2.4192 (13)
			
O1^i^—Ca1—O2^ii^	149.66 (5)	O1—Ca1—O2	56.90 (4)
O1^i^—Ca1—O2^iii^	111.93 (5)	O1—P1—O3	115.75 (8)
O2^ii^—Ca1—O2^iii^	95.90 (4)	O1—P1—O2	107.96 (8)
O1^i^—Ca1—O3^iv^	81.56 (5)	O3—P1—O2	114.40 (8)
O2^ii^—Ca1—O3^iv^	74.49 (4)	O1—P1—H1	109.0 (12)
O2^iii^—Ca1—O3^iv^	161.56 (5)	O3—P1—H1	104.1 (11)
O1^i^—Ca1—O3^v^	87.17 (5)	O2—P1—H1	104.9 (11)
O2^ii^—Ca1—O3^v^	89.43 (5)	P1—O1—Ca1^ii^	150.21 (8)
O2^iii^—Ca1—O3^v^	73.62 (4)	P1—O1—Ca1	99.88 (7)
O3^iv^—Ca1—O3^v^	121.07 (5)	Ca1^ii^—O1—Ca1	108.30 (5)
O1^i^—Ca1—O1	122.62 (4)	P1—O2—Ca1^i^	120.90 (7)
O2^ii^—Ca1—O1	73.14 (4)	P1—O2—Ca1^iii^	131.49 (8)
O2^iii^—Ca1—O1	78.87 (4)	Ca1^i^—O2—Ca1^iii^	105.94 (5)
O3^iv^—Ca1—O1	83.24 (5)	P1—O2—Ca1	91.01 (6)
O3^v^—Ca1—O1	145.62 (5)	Ca1^i^—O2—Ca1	99.78 (5)
O1^i^—Ca1—O2	70.38 (4)	Ca1^iii^—O2—Ca1	92.99 (5)
O2^ii^—Ca1—O2	130.00 (4)	P1—O3—Ca1^vi^	127.66 (7)
O2^iii^—Ca1—O2	77.54 (5)	P1—O3—Ca1^vii^	128.07 (7)
O3^iv^—Ca1—O2	96.39 (4)	Ca1^vi^—O3—Ca1^vii^	103.77 (5)
O3^v^—Ca1—O2	133.13 (5)		
			
O3—P1—O1—Ca1^ii^	11.3 (2)	O3—P1—O2—Ca1^iii^	53.71 (12)
O2—P1—O1—Ca1^ii^	140.99 (16)	O1—P1—O2—Ca1	18.17 (8)
O3—P1—O1—Ca1	−149.73 (6)	O3—P1—O2—Ca1	148.60 (6)
O2—P1—O1—Ca1	−20.05 (8)	O1—P1—O3—Ca1^vi^	78.88 (11)
O1—P1—O2—Ca1^i^	120.21 (8)	O2—P1—O3—Ca1^vi^	−47.62 (13)
O3—P1—O2—Ca1^i^	−109.36 (9)	O1—P1—O3—Ca1^vii^	−110.53 (10)
O1—P1—O2—Ca1^iii^	−76.72 (11)	O2—P1—O3—Ca1^vii^	122.98 (9)

Symmetry codes: (i) *x*−1/2, −*y*+1/2, −*z*+1/4; (ii) *x*+1/2, −*y*+1/2, −*z*+1/4; (iii) *y*, *x*, −*z*; (iv) *y*−1/2, −*x*+1/2, *z*+1/4; (v) *x*, *y*−1, *z*; (vi) −*y*+1/2, *x*+1/2, *z*−1/4; (vii) *x*, *y*+1, *z*.
