# Supplementary material for: Synthesis and crystal structure of calcium hydrogen phosphite, CaHPO3
Source: Acta Crystallogr E Crystallogr Commun. 2019 Jun 14;75(Pt 7):997–1000. doi: 10.1107/S2056989019008235 (PMC6659324; doi:10.1107/S2056989019008235)
Supplement: Supplementary file 3 [file e-75-00997-sup3.docx]

**Synthesis and crystal structure of calcium hydrogen phosphite, CaHPO_3_**

**Mark L. F. Phillips and William T. A. Harrison**

**Supplementary materials: X-ray powder pattern for CaHPO_3_ and IR spectrum of CaHPO_3_**

**
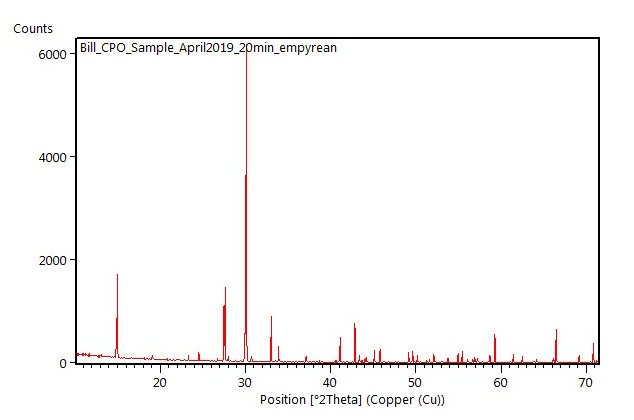
**

**
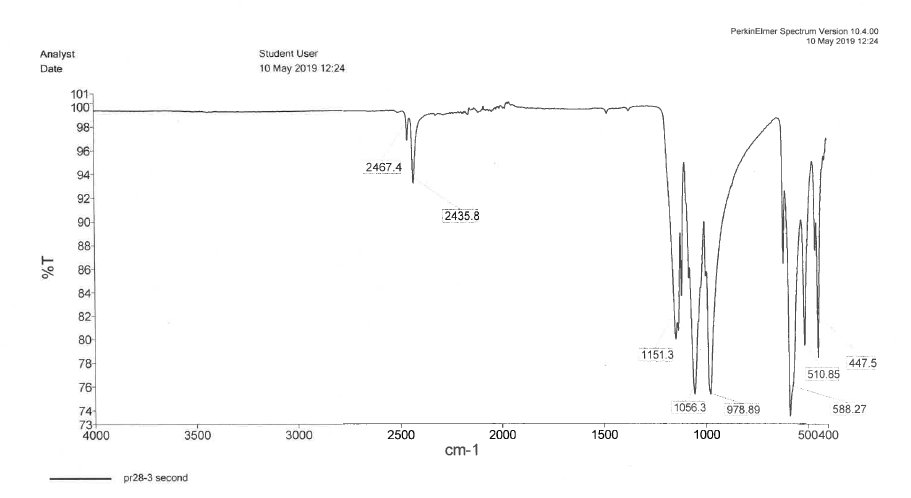
**
